# Phlegmasia Cerulea Dolens: A Rare Vascular Emergency

**DOI:** 10.7759/cureus.77678

**Published:** 2025-01-19

**Authors:** Lucas N Canaan, Daniel Whitson, Deepak Dev Vivekanandan, Nizar Hariri

**Affiliations:** 1 Surgery, Northeast Georgia Medical Center Gainsville, Gainesville, USA; 2 Vascular Surgery, Northeast Georgia Medical Center Gainsville, Gainesville, USA

**Keywords:** amputation, deep vein thrombosis (dvt), heparin-induced thrombocytopenia (hit), phlegmasia cerulea dolens, thrombectomy

## Abstract

Phlegmasia cerulea dolens (PCD) is a rare condition characterized by near-complete to total occlusion of an extremity, leading to discoloration, cyanosis, and pain caused by venous thromboembolism (VTE). The inciting events leading to VTE formation can vary widely and may include factors such as trauma or malignancy. In many cases, the underlying thrombus can be managed with anticoagulation. However, surgical intervention is required in certain situations, such as this patient. Prompt action is essential regardless of the treatment modality, as the underlying disease process can significantly threaten the affected limb. This pathology is associated with a high rate of amputation and mortality. This case report discusses a 69-year-old female with a thrombus affecting the common iliac, external iliac, femoral, popliteal, and tibial veins, resulting in PCD. It emphasizes the importance of urgent intervention and treatment and the severe consequences of this disease process.

## Introduction

Venous thromboembolism, including deep vein thrombosis (DVT) and pulmonary embolism (PE), is common in modern medicine. There is significant variability in the severity of presentation, ranging from relatively small DVTs that require no intervention to severe forms, such as phlegmasia cerulea dolens (PCD). PCD is a massive clot burden within the deep veins, particularly involving the more central veins [1]. DVT is a significant health problem in the United States and abroad. Although the exact number of affected individuals is unknown, estimates suggest up to 600,000 cases annually [2]. When considering the cascade of complications associated with DVT, many focus on the pathology of PE. While PE is more common, PCD is a life- and limb-threatening condition. It typically presents with swelling of the affected extremity, pain, and cyanosis. Although PCD results from venous outflow obstruction, it can progress to arterial ischemia due to a lack of forward flow [3]. Additional skin changes may occur as the disease advances, including bullae formation and skin necrosis [4].

There is variability in the management of PCD, and no gold standard therapy exists due to the rarity of the disease [5]. Regardless of the selected management, treatment must begin urgently. The mainstays of early treatment include preventing clot propagation, supportive measures such as elevation of the affected extremity and avoidance of hypovolemic shock, and treatment of the underlying condition [4]. In this case, the underlying condition was the patient's history of heparin-induced thrombocytopenia (HIT), which placed her in a hypercoagulable state. Since she had already failed outpatient management with warfarin, she was placed on argatroban in the emergency department. There is variability in surgical interventions as well. Multiple endovascular options can be deployed in addition to open thrombectomy. Endovascular therapy was selected for this patient; fortunately, thrombectomy was performed endovascularly. On some occasions, if swelling is significant, fasciotomies are required due to increased compartment pressures; however, this was not necessary for the patient referenced in this case study [3]. Throughout her hospital course, her left lower extremity compartments remained soft.

The pathogenesis of the disease begins with the occlusion or near-total occlusion of a large clot in a vein of the extremity. This leads to venous hypertension in the early stages and increased interstitial pressures as the disease progresses [6]. A hallmark of this disease is the occlusion of the main outflow, along with the collateral venous circulation becoming occluded [4]. As venous congestion worsens, an imbalance between the hydrostatic and oncotic pressures, which are normally balanced, occurs. The intravascular hydrostatic pressure increases, leading to fluid sequestration in the affected limb. In the severe forms of this disease process, if the compartment pressure becomes high enough, it can collapse the arterial walls, leading to acute ischemia and gangrene. This underscores the need to avoid hypovolemia and hemodynamic instability, as these would worsen the overall survivability of both the limb and the patient [4].

Given the rarity of this disease process, few large studies have been conducted. Much of the medical literature on PCD comprises case reports [1,6]. Overall, the prognosis is grim, with a mortality rate ranging from 20% to 40%. Amputations of the affected limb are also common, with an amputation rate ranging from 20% to 50% [4,5]. This again underscores the importance of early recognition and treatment of this devastating disease.

## Case presentation

**Figure 1:**
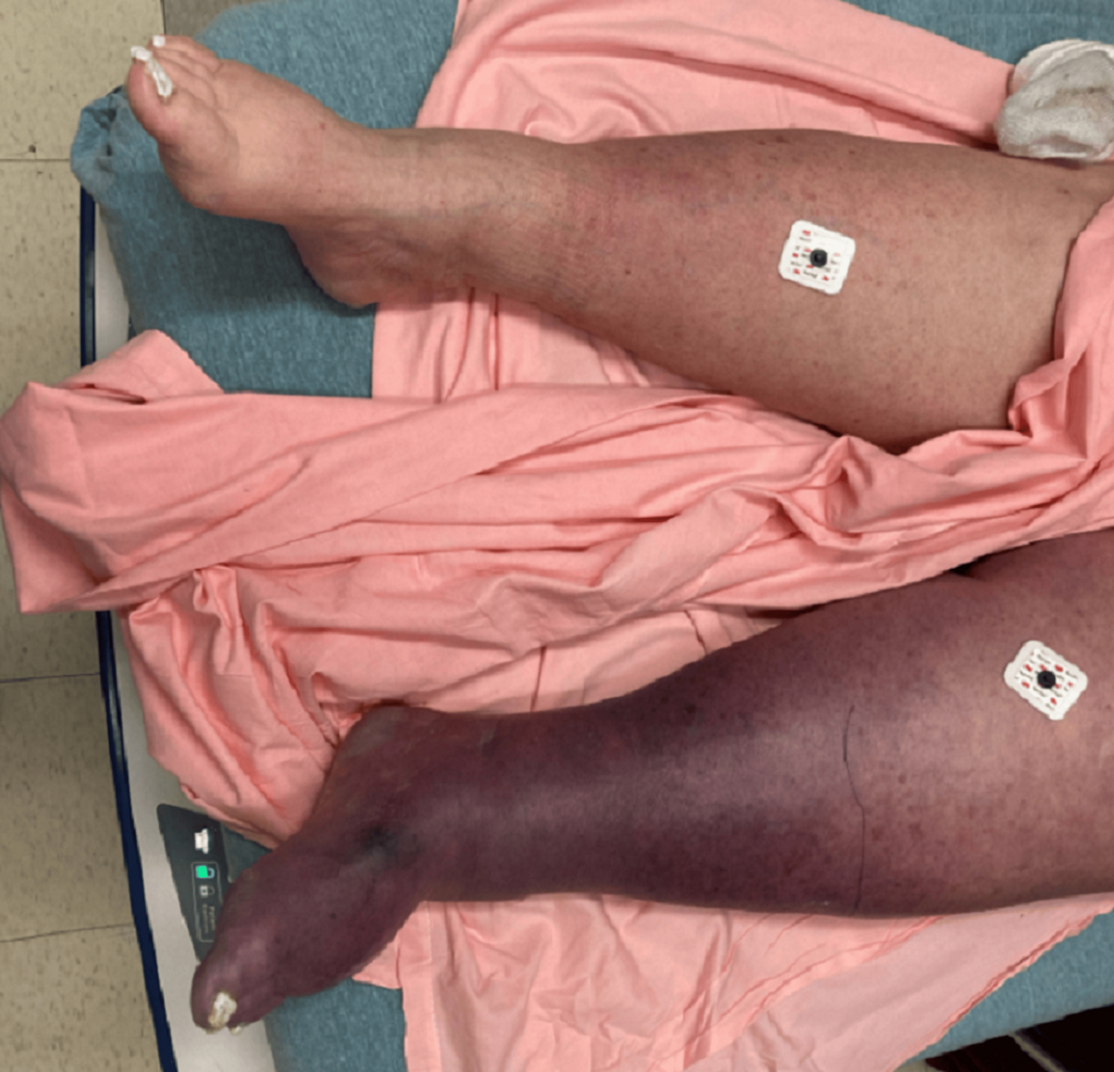
Swelling and discoloration of the patient’s left leg on admission to the hospital prior to surgical intervention

A 69-year-old female with a past medical history significant for heart failure with preserved ejection fraction and morbid obesity presents to the emergency department with acute lower limb pain that began the night before and worsened through the morning. Her primary complaints include swelling, coolness in her left foot, discoloration, and cyanosis, as seen in Figure 1. The vascular surgery team was consulted due to concerns about acute limb ischemia or a “cold leg.” The patient was discharged from the hospital approximately two days prior after being treated for acute kidney injury and HIT. HIT was suspected after her platelets dropped significantly from 177 to 97, ultimately decreasing to 55. She tested positive for HIT antibodies and had a positive serotonin release assay. All heparin-containing products were discontinued, argatroban was initiated, and she was bridged to warfarin, which was the medication she was ultimately discharged on. Prior to discharge, she was evaluated for DVT with a venous duplex, which revealed no DVT in her lower extremities. Upon returning to the hospital, her platelet levels began to recover slowly to 74, and she was noted to have a supratherapeutic INR. The remainder of her admission lab work can be seen in Table 1.

**Table 1:** Lab values on admission

Lab	Value	Nominal ranges	Units
White blood cell	17.2	4.8-10.8	K/µL
Hemoglobin	13.7	12.0-16.0	g/dL
Hematocrit	42.0	37.0-47.0	%
Platelets	74	130-400	K/µL
Sodium	131	135-148	mmol/L
Potassium	4.1	3.5-5.2	mmol/L
Chloride	101	100-110	mmol/L
CO2	19	21-32	mmol/L
Blood urea nitrogen	20.0	5.0-32.0	mg/dL
Creatinine	1.90	0.60-1.00	mg/dL
Glucose	132	65-99	mg/dL
International normalized ratio	3.39	0.87-1.14	-
Prothrombin time	39.8	9.4-12.5	Seconds
partial thromboplastin	46.5	25.1-36.5	Seconds

At the initial evaluation by the vascular surgery team, the dorsalis pedis and posterior tibial pulses were easily palpable in the right lower extremity but significantly more challenging in the left. Doppler signals were present for both arteries in the left lower extremity. The patient denied any sensory deficits and could move her left foot on command. Based on the initial clinical presentation, there was significant concern for venous outflow obstruction.

**Figure 2:**
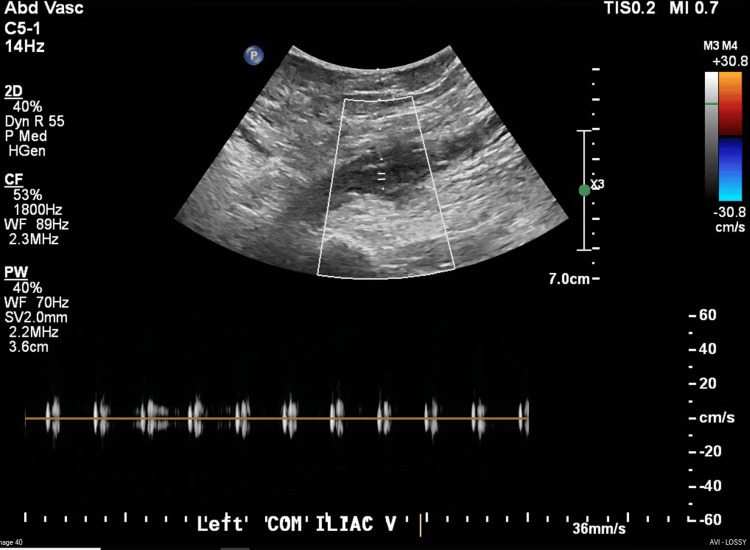
Duplex of the left common femoral vein demonstrating non-compressible thrombus and no flow within the area of the white box

Work-up up to that point did not reveal any significant lab abnormalities in the complete blood count or comprehensive metabolic profile; however, she did have a supratherapeutic INR and increased creatinine. CT angiography of her left lower extremity showed three-vessel runoff. A venous duplex of her left leg demonstrated acute thrombus within the left common femoral vein, left saphenofemoral junction, left greater saphenous vein, left deep femoral vein, and left superficial femoral vein, consistent with her exam findings. The duplex of the left common iliac vein is shown in Figure 2. Based on the physical exam and history, in conjunction with her workup in the emergency department, the most likely diagnosis is PCD.

**Figure 3:**
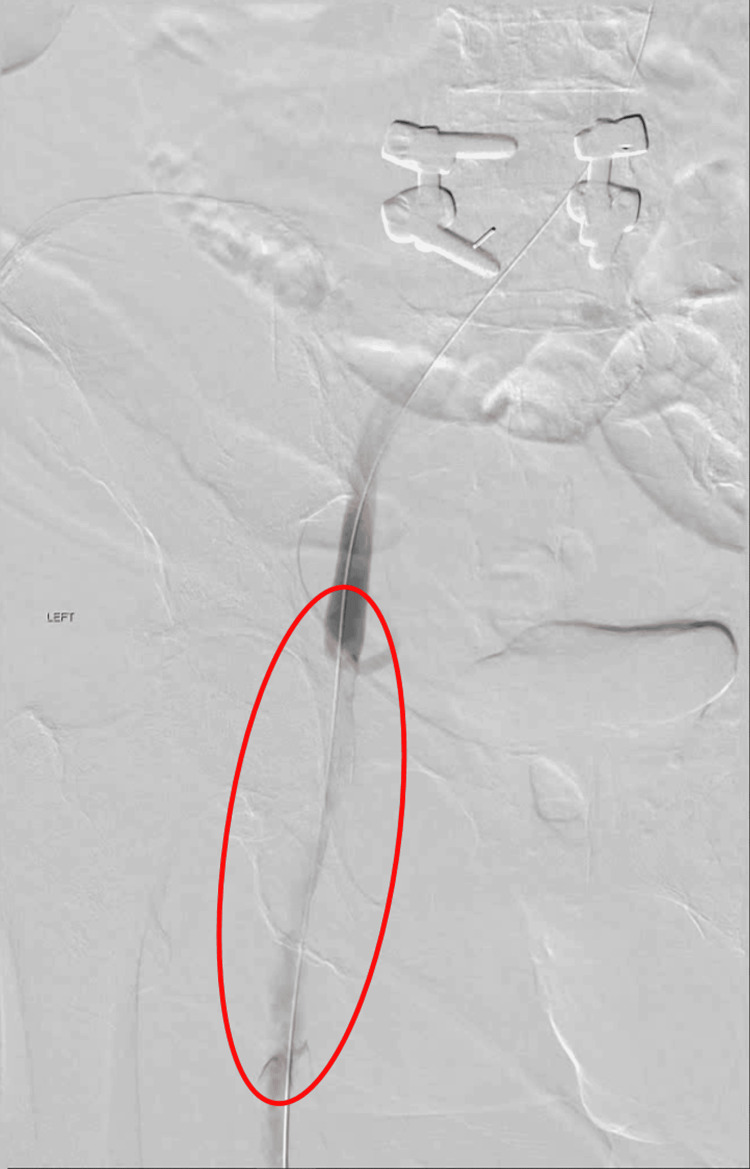
Initial venogram showing the occlusion of the external iliac and common iliac veins (red circle) which is consistent with VDS findings VDS: venous duplex scan

**Figure 4:**
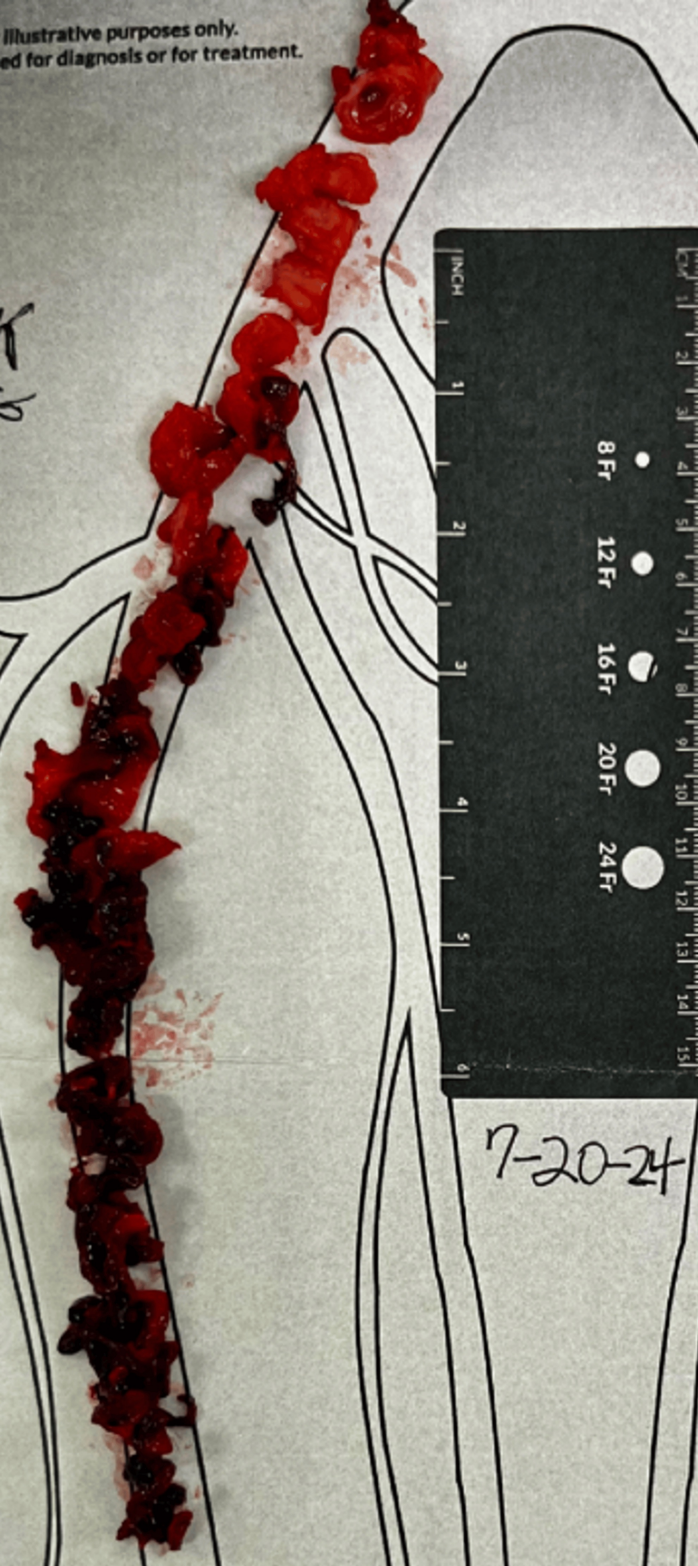
Thrombus removed after suction thrombectomy with chronic clot noted at the top of the picture and more acute clot noted toward the bottom

**Figure 5:**
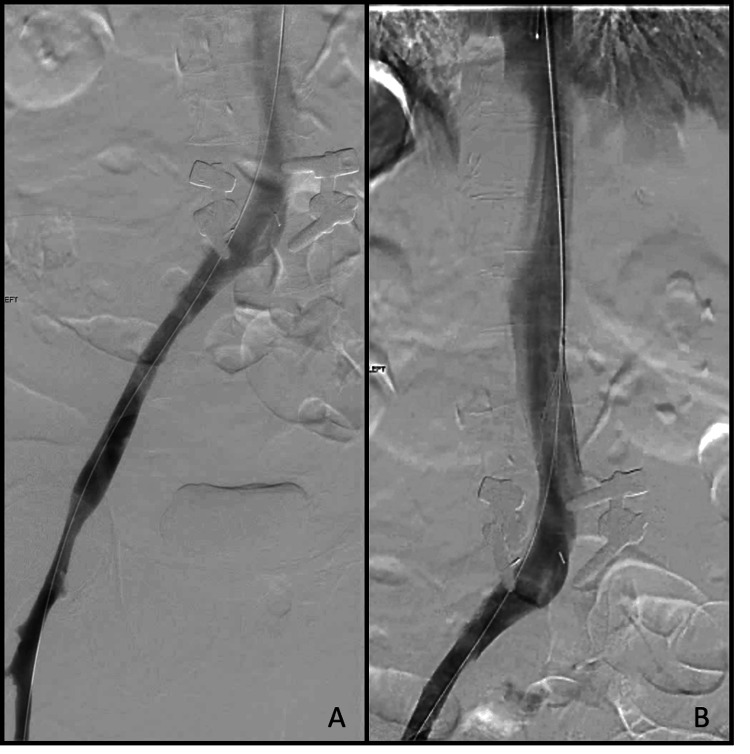
Venogram after thrombectomy showing good flow and decreased clot burden in the external and common iliac veins (A) as well as good contrast flow through the IVC (B) IVC: inferior vena cava

With the patient having HIT and the inability to start heparin, it was decided to begin her on argatroban, a direct thrombin inhibitor. She was emergently taken to the endovascular operating room for a thrombectomy. The approach selected for thrombectomy was a percutaneous mechanical thrombectomy from a posterior popliteal vein as the access site. Access was gained using a micro-puncture kit. Due to the patient's swelling and body habitus, the access needle was not long enough. A spinal needle was required to access the popliteal vein, followed by the placement of a 4 French micropuncture sheath. A venogram was completed to confirm the correct position, followed by advancing the guide wire into the inferior vena cava (IVC). The venogram also confirmed the findings on the venous duplex and demonstrated occlusion of the left common iliac vein and left external iliac vein, as seen in Figure 3. The 4 French micropuncture sheath was upsized to a 13 French sheath to facilitate mechanical thrombectomy. The Inari suction thrombectomy device with the basket was passed multiple times to clear the affected area, removing both acute and chronic clots (Figure 4). A completion venogram showed restored patency of the affected area, as seen in Figure 5. Finally, an IVC filter was placed prior to the completion of the procedure, as the patient had already had a failed outpatient therapy on warfarin, even in the setting of supratherapeutic INR. The patient was then taken to the medical ICU for neuro checks and pulse exams.

Outcome

**Figure 6:**
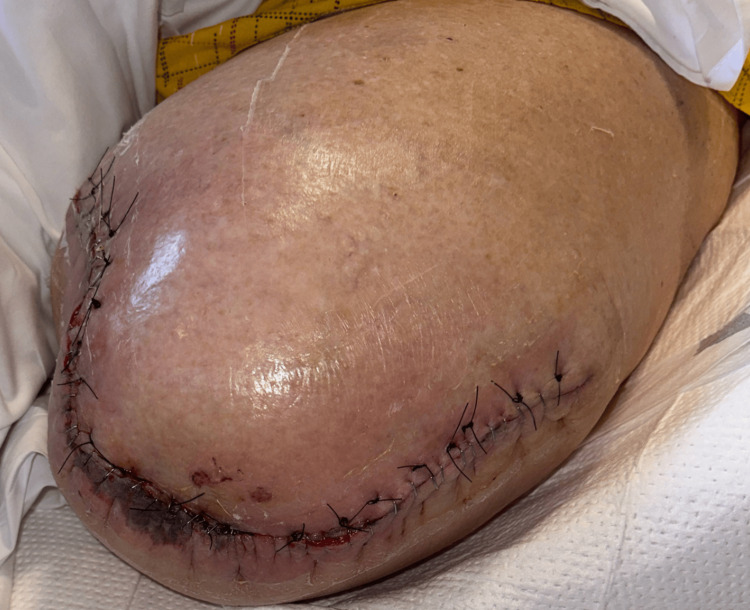
Left AKA stump during the postoperative course after significant non-viable soft tissue loss AKA: above-knee amputation

She had a largely uneventful post-procedural course, with the most significant issue being pain control. During her hospital stay, she developed an acute kidney injury that did not require dialysis and ultimately improved with gentle hydration using isotonic fluids. Over the following days, the left lower extremity did not improve and became demarcated. A discussion was held with the patient regarding her condition and the likely nonviable nature of her left lower extremity, with amputation being proposed. After much deliberation, the patient agreed to this plan. She then proceeded to the operating room for a left above-knee amputation (AKA) with the vascular surgery team. Due to edema and her body habitus, the amputation was technically challenging, with the primary concern being the dehiscence of the stump due to tension. The final result of her left AKA is shown in Figure 6. During her hospital course, there were no issues postoperatively from the AKA, and it is healing well. The initial result of the AKA is also depicted in Figure 6. Throughout her hospital stay, she worked with physical therapy, which revealed some difficulties following the amputation. Given her multiple health issues, she was sent to rehab at discharge for further strengthening.

## Discussion

The diagnosis and treatment of this 69-year-old female not only highlight the rarity of PCD but also emphasize the overall grim nature of the disease. She presented classically to the emergency department with a history and physical exam consistent with PCD. The patient's delayed presentation to the hospital likely contributed to the severity of her condition, ultimately leading to her amputation, as her symptoms and findings were more advanced. The literature does not describe a specific treatment protocol for this disease process; however, the patient's care proceeded in a manner consistent with available case studies and recommendations, including anticoagulation with argatroban and urgent thrombectomy [2,3,5,6].

This patient's course followed a similar pattern to the currently available literature. However, no studies were significantly sized, as the incidence of this disease process is relatively rare. With the patient's initial presentation being delayed and the prominent skin changes and swelling present, this increased her preoperative risk of amputation as part of her hospital course. As consistent with the literature, the first priority of management is anticoagulation, followed by urgent thrombectomy [2,3,5,6]. In evaluating anticoagulants, argatroban is a common selection, as it has a relatively short half-life, making it ideal for patients who might need subsequent procedures. It is also a direct thrombin inhibitor, considered first-line in HIT anticoagulation [7]. Regarding thrombectomy, there are two broad categories for management: open and percutaneous. Given the patient's body habitus and significant swelling, a percutaneous approach was chosen. Both open and percutaneous approaches are well documented in the literature, with open approaches being considered "older" and percutaneous approaches being more preferred in the modern era [8]. For open approaches, the vein or a distal segment of the affected vein is directly accessed, and a Fogarty balloon is used to remove the clot from the vessel. The vessel is then primarily closed or patched if needed. Regarding percutaneous approaches, access can be accomplished from various locations, including the internal jugular vein, common femoral vein, or popliteal vein [9]. The specific site depends on the comfort and preference of the attending surgeon and the preference for devices to use. Once access is gained, either pharmacotherapy or mechanical thrombectomy can be performed [8]. In the case of this patient, a mechanical thrombectomy was performed with good results.

This case offers a stark reminder of the importance of early patient evaluation. The initial consult with the vascular surgery team indicated critical limb ischemia and suspected PCD. Although the team's response was prompt, significant tissue damage had already occurred by the time of initial evaluation. Her recent history of HIT further complicated the patient's clinical course. Excluding other disease processes, HIT carries its own morbidity. Despite platelet reduction, HIT places patients in a prothrombotic state, which likely contributes to the underlying pathology of the patient's presentation, as the most common complication of HIT is thrombosis [7]. In one study, nearly 30% of patients diagnosed with HIT developed thrombosis [10].

The patient's diagnosis of HIT during her prior hospitalization certainly contributed to her development of PCD. Either pathology alone can be devastating. The delayed presentation to the emergency department contributed to her poor overall outcome, including the need for amputation.

## Conclusions

PCD is a rare thrombotic process with severe outcomes, including the potential loss of a limb or even life. Treatment must begin early to reduce the overall poor outcomes. High-level management includes supportive care while avoiding hemodynamic instability or hypotension, elevating the affected extremity, preventing propagation, and treating the underlying cause. In select patients, such as the one presented here, surgical intervention is warranted, which may include either endovascular thrombectomy or open thrombectomy.
